# Coronary Artery Disease and Revascularization in Patients Undergoing Transcatheter Aortic Valve Replacement

**DOI:** 10.31083/j.rcm2309290

**Published:** 2022-08-22

**Authors:** Aakash Garg, Suleman Ilyas, Michael Cryer, J. Dawn Abbott

**Affiliations:** ^1^Division of Cardiology, Warren Alpert Medical School of Brown University, Providence, RI 02903, USA; ^2^Division of Cardiology, Tucson Medical Center, Tucson, AZ 85712, USA

**Keywords:** aortic stenosis, coronary artery disease, percutaneous coronary intervention, transcatheter aortic valve replacement

## Abstract

Coronary artery disease (CAD) and aortic stenosis share similar risk factors and underlying pathophysiology. Up to half of the patient population undergoing work-up for aortic valve replacement have underlying CAD, which can affect outcomes in patients with more severe disease. As the indications for transcatheter aortic valve replacement (TAVR) have expanded to intermediate and now low risk patients, the optimal management of CAD in this patient population still needs to be determined. This includes both pre-TAVR evaluation for CAD as well as indications for revascularization in patients undergoing TAVR. There is also limited data on coronary interventions after TAVR, including the incidence, feasibility and outcomes of patients undergoing percutaneous coronary intervention (PCI) after TAVR. This review provides an updated report of the current literature on CAD in TAVR patients, focusing on its prevalence, impact on outcomes, timing of revascularization and potential challenges with coronary interventions post-TAVR.

## 1. Introduction

Aortic stenosis (AS) is an insidious disease with an asymptomatic course followed by a gradual decline after the presentation of symptoms [1]. Coronary artery disease (CAD) is common in patients with AS, likely due to overlapping risk factors, with prevalence as high as 60% [2]. In the last decade, transcatheter aortic valve replacement (TAVR) has revolutionized the management of severe symptomatic AS and is now the preferred treatment modality in patients at intermediate and high surgical risk [3]. For surgical aortic valve replacement (SAVR), the standard of care has been concomitant revascularization via bypass grafting during the time of valve replacement. Similarly, guidelines recommend that percutaneous coronary intervention (PCI) of significant lesions is reasonable among patients undergoing TAVR [4, 5]. However, the prognostic impact of CAD and its optimal management in such patients remain a matter of debate. Further, as the indication of TAVR expands to include younger and lower-risk patients, with risk of development or progression of CAD, many of these patients may need coronary angiography and revascularization after TAVR. As such, there is also paucity of data regarding optimal timing of revascularization among patients planned for TAVR as well as the feasibility and outcomes of patients requiring PCI after TAVR. The aims of this review are (1) describe the prevalence of CAD and its prognostic impact among TAVR patients, (2) analyze the role of invasive coronary physiology and computed tomographic angiography (CTA) in the evaluation of CAD pre-TAVR, (3) summarize the data on timing of PCI among patients undergoing TAVR, and (4) discuss the management of CAD after TAVR including potential challenges with coronary access.

## 2. Prevalence of CAD 

Originally thought to be a disease of “wear and tear” and age-associated valvular degeneration, AS is now known to share a similar pathophysiological mechanism to atherosclerosis, beginning with endothelial damage secondary to increased mechanical and reduced shear stress followed by the infiltration of lipid and inflammatory cells into the subendothelial space, resulting in disorganized fibrous tissue deposition and further inflammatory cell recruitment [6, 7]. Many of the same risk factors for CAD are also involved in the pathogenesis of severe calcific AS, including hypertension, dyslipidemia, cigarette smoking, diabetes mellitus, and advancing age.

The prevalence of CAD in patients with severe symptomatic AS has been estimated to be between 40–75% depending on the definition used [2, 3]. TAVR clinical trials highlight the important differences in CAD prevalence among different patient populations (Table 1) [8, 9, 10, 11, 12, 13, 14, 15]. In the original PARTNER 1A and 1B study cohorts, 74.9% of the 348 patients and 67.6% of the 179 patients undergoing TAVR had history of CAD, respectively [8, 9]. Similarly, the prevalence rates of CAD were 75.5–81.8% in the CoreValve US Extreme Risk and High Risk trials [10, 11]. Furthermore, in the intermediate-risk trials (PARTNER 2 and SURTAVI), ~60% of patients had CAD [12, 13]. Whereas, the prevalence of CAD was much lower at 15–27.7% in the low-risk trials (PARTNER 3 and Evolut Low risk trial) [14, 15]. Real world registry studies have also provided useful insights [16, 17, 18, 19, 20, 21, 22, 23]. In the FRANCE 2 registry of 3195 patients undergoing TAVR, 47.9% had CAD [16]. Moreover, multivessel disease was found in approximately one-half of patients undergoing PCI before TAVR in another multicenter study [24].

**Table 1.** **Coronary artery disease and outcomes in major TAVR randomized controlled trials**.

Trial name	First author, year (Ref. #)	CAD	Prior MI	Prior CABG	Prior PCI	STS Score	30-Day/In hospital MI	1-YR MI	30-Day/In hospital death	1-YR death
PARTNER 1A	Smith *et al*., 2011 [8]	74.9%	26.8%	42.6%	34%	11.8 ± 3.3	0%	0.4%	3.4%	24.2%
n = 348										
PARTNER 1B	Leon *et al*., 2010 [9]	67.6%	18.6%	37.4%	30.5%	11.2 ± 5.8	0%	0.6%	5%	30.7%
n = 179										
CoreValve US Extreme Risk	Popma *et al*., 2014 [10]	81.8%	30.9%	39.5%	37%	10.3 ± 5.5	1.2%	2.0%	8.4%	24.3%
n = 489										
CoreValve US High Risk	Adams *et al*., 2014 [11]	75.4%	25.6%	29.7%	33.8%	7.3 ± 5.8	0.8%	1.9%	3.3%	14.2%
n = 394										
PARTNER 2	Leon *et al*., 2016 [12]	69.2%	18.3%	23.6%	27.1%	5.8 ± 2.1	1.2%	2.5%	3.9%	12.3%
n = 1011										
SURTAVI	Reardon *et al*., 2017 [13]	62.6%	14.5%	16%	21.3%	4.4 ± 1.5	0.9%	2.0%	2.2%	6.7%
n = 864										
PARTNER 3	Mack *et al*., 2019 [14]	27.7%	5.7%			1.9 ± 0.7	1%	1.2%	0.4%	1%
n = 496										
Evolut Low Risk	Popma *et al*., 2019 [15]		6.6%	2.5%	14.2%	1.9 ± 0.7	0.9%	1.7%	0.5%	2.4%
n = 725										

CAD, coronary artery disease; MI, myocardial infarction; CABG, coronary artery bypass grafting; PCI, percutaneous coronary intervention; STS Score, Society of Thoracic Surgeons Risk of Mortality provides an estimate of the risk of death at 30 days among patients undergoing surgical aortic-valve replacement on the basis of several demographic and procedural variables.

## 3. Impact of CAD on Outcomes 

The presence of CAD has been known to increase the risk for periprocedural complications in patients undergoing SAVR and affects long-term clinical outcomes as well [25]. However, the studies evaluating impact of CAD on patients undergoing TAVR have yielded conflicting results [26, 27, 28, 29, 30]. Ussia *et al*. [26] evaluated the impact of CAD, defined as previous PCI or surgical revascularization, among 663 consecutive patients who underwent TAVI at 14 institutions across Italy. The authors found that patients with CAD had similar one-year rate of major adverse cardiovascular and cerebral events (MACCE) compared with those without CAD (CAD group vs no-CAD group, 15.7% vs 18.3%; adjusted hazard ratio [HR] 0.76; 95% confidence interval [CI] 0.42 to 1.36; *p* = 0.353). On the contrary, CAD was associated with worse outcomes in the study by Franzone *et al*. [27]. In this study of 496 patients undergoing TAVR, patients with concomitant CAD had increased risk of MACCE compared to patients without CAD (HR 1.75, 95% confidence interval, CI, 1.06–2.89). Two meta-analyses looking at prognostic significance of CAD among TAVR patients have also showed contrasting results [28, 29]. D’Ascenzo *et al*. [28] in their meta-analysis of adjusted outcomes from 7 observational studies (2472 patients) reported no association between presence of CAD and mortality at 1 year follow-up. Whereas, another meta-analysis of 15 studies with 8013 patients showed a significant increase in 1-year all-cause mortality in the CAD group compared to patients without CAD {Odds ratio (OR) 1.21 (95% CI 1.07–1.36); *p* = 0.002} [29]. Of note, the anatomic extent and complexity of CAD was not systematically assessed in these studies. Other reasons for discrepant results include (1) variability in CAD definition across studies with few studies using only prior revascularization and others using stenosis of either >50% or >70%, (2) heterogeneity in management and revascularization of significant CAD prior to TAVR, and (3) limited follow-up period (1–2 years) in most studies.

## 4. SYNTAX Score and Outcomes 

Several studies have quantified CAD burden with the SYNTAX score (SS) as an objective measure of CAD severity in evaluating link with TAVR outcomes [31, 32, 33, 34, 35, 36]. In the study by Stefanini *et al*., baseline and residual SS were determinants of adverse outcomes. At baseline, a score of >22 was associated with worse cardiovascular mortality compared to 22 or lower. and no CAD (20.4% vs 13.6% vs 8.6%, respectively; *p* = 0.029) [31]. In patients who underwent revascularization, residual SS (>14) was similarly associated with worse outcomes. Consistent findings have been observed in other studies looking at relationship between SS and post-TAVR outcomes [32, 33]. Further, Stephan *et al*. [34] found that patients with concomitant CAD suffered more frequently from myocardial infarction (MI) post-TAVR, and that patients with a residual SS <8 showed significantly lower rates of one-year mortality. In these studies, extent and severity of CAD frequently correlated with higher comorbidity burden and greater risk profiles at baseline [3]. Although the limitations of observational nature of these studies need to be accounted, altogether these findings may suggest an association between CAD severity, completeness of revascularization, and impaired ischemic outcomes post-TAVR.

## 5. Assessment of CAD

### 5.1 Role of Invasive Physiology 

Fractional flow reserve (FFR) and non-hyperemic pressure ratios such as instantaneous wave-free ratio (iFR) are routinely utilized for functional assessment of intermediate coronary stenoses and to guide revascularization [37]. However, these indices have not been validated in patients with severe AS. Left ventricular hypertrophy induced by AS may alter coronary flow reserve and thus FFR may theoretically underestimate the degree of coronary stenosis in patients with severe AS. Few studies have demonstrated the safety of adenosine bolus and examined the role of FFR in the pre-TAVR patient population [38]. Ahmad *et al*. [39] studied coronary flow indices in 28 patients pre and post-TAVR and found that systolic hyperemic flow significantly increased and FFR significantly decreased post-TAVR, suggesting underestimation of coronary stenosis severity with FFR. Conversely, there was no change in flow during the wave-free diastolic period and iFR remained similar pre and post-TAVR. Further, in another study evaluating 54 patients with 133 coronary lesions with severe AS before and after TAVR, FFR variations after TAVR were minor compared with baseline measurements [40]. However, FFR values tended to worsen after TAVR in patients with pre-TAVR positive FFR (<0.80) and those with >50% angiographic stenosis thus suggesting that FFR post-TAVR may unmask physiological significance of certain intermediate coronary lesions. Scarsini *et al*. [41] evaluated a hybrid iFR-FFR decision making strategy. Using an iFR value of >0.93, had a 98.4% negative predictive value for a non-significant FFR, but an iFR <0.83 only had a positive predictive value of 91.3% for a significant FFR (≤0.80). Yamanaka *et al*. [42] demonstrated excellent reproducibility of iFR and good correlation between FFR and iFR in patients with severe AS. Their work also indicated that iFR cutoff value of 0.82 correlated well with FFR <0.75 and presence of reversible myocardial perfusion defects in patients with AS. Overall, these studies demonstrate safety and feasibility of physiological indices in CAD evaluation among patients undergoing pre-TAVR coronary angiography, but further study is needed to determine the appropriate cut-off values for ischemia. Ongoing randomized controlled trials (RCTs) [FAITAVI (NCT03360591), NOTION 3 (NCT 03058627)] will inform the role of physiology-guided revascularization in patients undergoing TAVR.

### 5.2 Role of CTA 

Gated CTA is the modality of choice for evaluation of aortic annular sizing and vascular access in patients planned for TAVR. As in patients without AS, CTA has been studied for evaluation of CAD among patients awaiting TAVR [43, 44, 45, 46]. Van den Boogert and colleagues examined the DEPICT CTA database to assess the diagnostic yield and accuracy of pre-TAVR CTA, as compared with coronary angiography, to detect significant left main and proximal coronary stenosis [43]. Their analysis of 1060 patients revealed that the CTA excluded proximal coronary stenosis with a sensitivity of 96.4% and NPV of 98.0% for a threshold of ≥50%, and a sensitivity of 96.7% and NPV of 99.0% for a threshold of ≥70% diameter stenosis. They concluded that based on the threshold of 50% or 70% diameter stenosis, the need for coronary angiography would decrease in TAVR patients by 52% or 70% respectively. A systematic review and meta-analysis on the diagnostic accuracy of coronary CTA to detect CAD in patients referred for TAVR (7 studies, n = 1275) resulted in a sensitivity, specificity, PPV and NPV of 95.3%, 65.3%, 70.8%, and 94.0% respectively [44]. An ongoing RCT [CT-CA (NCT 03291925)] will prospectively compare CTA-based selective coronary angiography vs routine angiography in patients planned for TAVR. Decreasing the need of coronary angiography, particularly in elderly frail patients, can potentially reduce contrast exposure, vascular complications, and healthcare costs.

## 6. Timing of PCI in Patients Undergoing TAVR

About 12% of TAVR patients included in RCTs underwent PCI before TAVR, and this increased to ~25% in TAVR registry studies [3]. Although the guidelines suggest PCI for proximal coronary stenosis >70% in patients undergoing TAVR, clinical importance and optimal timing remains unclear [4, 5]. PCI can be performed either before TAVR, concomitantly with TAVR or be staged after TAVR. There are several considerations regarding the safety and feasibility of each approach.

Table 2 highlights several studies comparing outcomes of patients undergoing TAVR with concomitant PCI versus TAVR alone [47, 48, 49, 50, 51].

**Table 2.** **Studies examining outcomes of TAVR with or without PCI**.

First author (Ref. #)	CAD	Logistic EuroScore	30 day mortality	Follow-up mortality	Summary
Guedeney *et al*., 2019 [47]				1-year	Increased 1-year mortality in PCI group compared with no CAD group
81 TAVR + PCI	100%	17.7	6.20%	17.30%	
247 TAVR alone (CAD)	100%	19	3.60%	13.90%	
459 TAVR alone (no CAD)	0%	16.6	3.50%	10.10%	
			*p* = 0.29	*p* = 0.11	
Abdel-Wahab *et al*., 2012 [48]				6 months	No difference in 6-month outcomes
55 TAVR + PCI (majority staged before TAVR)	100%	25.1	2%	9%	
70 TAVR alone	51.40%	23.6	6%	14%	
			*p* = 0.27	*p* = 0.42	
Wenaweser *et al*., 2011 [49]					Similar mortality at 30 days with PCI (staged or concomitant)
59 TAVR + PCI (staged or concomitant)	100%	26.8	10.20%	NR	
197 TAVR alone	54.80%	24.2	5.60%	NR	
			*p* = 0.24		
Conradi *et al*., 2011 [50]	100%	26.8	7.10%	NR	Higher risk of renal failure in concomitant approach
21 Staged PCI + TAVR					
7 Concomitant TAVR + PCI					
Millan-Iturbe *et al*., 2018 [51]		13.9	NR	9-year	No differences in 9-year all-cause mortality
136 TAVR + PCI	100%			55.10%	
88 isolated TAVR (with CAD)	100%			38.90%	
720 isolated TAVR (without CAD)	0%			45.70%	
				*p* = 0.23	

TAVR, transcatheter aortic valve replacement; PCI, percutaneous coronary intervention; CAD, coronary artery disease; NR, not reported; EuroScore, European system for cardiac operative risk evaluation.

### 6.1 PCI before TAVR

The pros of PCI prior to TAVR are (1) simplified coronary access without a prosthetic valve in place, and (2) less risk of hemodynamic instability and ischemia that may result from significant proximal coronary stenosis during rapid pacing for TAVR. The safety of PCI in patients with severe AS prior to TAVR has been demonstrated in several studies [48, 52, 53, 54]. Chakravarty *et al*. [52] demonstrated that planned PCI of the left main coronary artery among TAVR patients is feasible and safe with similar mortality compared to a matched cohort undergoing TAVR alone. Another large multi-center observational study sought to analyze the procedural features and late outcomes of pre-TAVR PCI among 1197 patients [24]. The mean SS was 12.1 ± 9.1, and PCI of left main and left anterior descending accounted for 9.3% and 37.6% of coronary lesions, respectively. Over a median follow-up of two years, target vessel failure was low (3.3%), however, 37.1% of patients sustained a MACCE. In a meta-analysis of 9 studies, PCI before TAVR was associated with similar 1-year mortality but there was a higher risk of major vascular complications [OR 1.86; 95% CI 1.33–2.60] compared to TAVR only group [53].

The ACTIVATION trial is the only prospective RCT that has prospectively compared outcomes of pre-TAVR PCI versus TAVR-only approach in patients with significant CAD [55]. The inclusion criteria were patients with TAVR eligible-severe AS, and CAD amenable to PCI. Patients were excluded if they had presented with ACS, had known angina of CCS 3 or greater, unprotected left main disease or a contraindication to dual antiplatelet agents. Overall, 235 patients were randomized; 119 patients assigned to undergo PCI and 116 underwent no PCI. At 1 year, there was no difference in mortality between the two groups, however an increase in bleeding events was observed in the PCI arm. Although widely quoted, one of the major limitations of this trial was slow recruitment and less than anticipated sample size. Furthermore, patients with left main disease or >CCS II angina were excluded, therefore the findings are not applicable to these subgroups. Future RCT with anatomy severity (for ex. SYNTAX score) and physiological assessment of CAD will further inform our practice regarding management of these patients.

### 6.2 Concomitant PCI and TAVR

The benefits of PCI combined with TAVR during same procedure may include reduction of vascular complications, less risk of hemodynamic instability and ischemia during TAVR and a theoretical reduction in mortality while waiting for TAVR. However, drawbacks include the increased contrast load and procedural time. In a study comparing concomitant PCI plus TAVR with staged PCI followed by TAVR, the authors found a statistically non-significant trend toward higher prevalence of major access-related complication and life-threatening bleeding in the staged PCI and TAVR group, whereas another study showed higher prevalence of acute renal injury in patients undergoing PCI and TAVR during the same setting [49, 50]. Currently, the practice of concomitant PCI and TAVR is uncommon.

### 6.3 Ongoing RCTs of Coronary Revascularization in AS

In the RCTs comparing TAVR and SAVR, patients with complex CAD were largely excluded.

The NOTION-3 trial (**NCT03058627**) aims to examine the role of FFR-guided complete revascularization compared to TAVR-only approach among patients with severe AS selected for TAVR and at least one coronary stenosis with FFR ≤0.80 or a diameter stenosis >90% in a coronary artery ≥2.5 mm. The TransCatheter Valve and Vessels Trial (TCW) is a noninferiority trial that is comparing a SAVR+CABG strategy to TAVR+FFR-guided PCI strategy among patients eligible for both TAVR or SAVR (**NCT03424941**). Results of these two studies will enhance our decision-making in patients with an indication for AVR and severe CAD.

## 7. Coronary Revascularization after TAVR 

Patients undergo PCI after TAVR implantation for a variety of reasons such as planned staged intervention, acute closure or disruption of the aorta-ostial vessels, acute coronary syndrome (ACS), and progression of stable CAD. In addition to traditional risk factors, there are unique characteristics that may increase the prevalence of coronary ischemia in patients post TAVR implant. These include disruption of coronary flow dynamics after bioprosthetic valve implantation which may lead to coronary hypoperfusion, coronary embolization from bioprosthetic valve thrombus formation, delayed migration of the valve leading the ostial coronary impingement, and even hypersensitivity reactions to the metallic anions in the valve frame known as Kounis syndrome [56, 57, 58, 59, 60]. In this section we will review the incidence, outcomes, and particular challenges associated with patients undergoing PCI post TAVR implant.

### 7.1 Incidence of Revascularization After TAVR

Early registry studies reported a varying incidence of PCI after TAVR ranging from 1–25.8% with success rates ranging from 85.7–100% [61, 62, 63, 64]. These studies were predominantly single center and with a self-expanding valve platform, limiting broad applicability. More recently, a multinational European registry including 15,325 patients evaluated a larger scope of valve types and observed an incidence of unplanned PCI post TAVR of 0.9% [65]. The median time to PCI was 191 days but the highest daily incidence was exhibited during the first week post implant. ACS made up roughly 60% of the cohort with the rest made up of stable coronary heart syndromes.

Vilalta *et al*. [66] showed a ~10% incidence of ACS after TAVR at a median follow-up of 25 months in a single center prospective registry of 779 patients. The distribution of ACS was as follows: 35.9% type 2 myocardial infarction (MI); 34.6% unstable angina; 28.2% non- ST elevation MI type I; and 1.3% ST-elevation MI. While most patients underwent revascularization with PCI, not all cases went to the catheterization lab, especially the non-stemi type II cohort of which the majority were managed medically.

In a multinational registry of patients who received LM PCI in relation to TAVR, PCI most often occurred prior to or at the time of TAVR [52]. However, 4.6% of patients underwent PCI of the LM emergently (within 24 hours) after implant. Most of these cases were the result of a TAVR related complication. This subset of patients demonstrated increased rates of cardiogenic shock, cardiac arrest, and mortality. Another 4.6% of patients underwent PCI of the LM post TAVR (beyond 24 hours after implant) with a median of 368 days post procedure. In this subgroup all cases were attributed to progression of LM CAD and there were no reports of difficulty in catheter engagement.

### 7.2 Outcomes of Patients Undergoing PCI After TAVR

Registry data provide insight into the incidence and procedural success of PCI post TAVR. Unfortunately, due to heterogeneous indications for PCI post TAVR, outcomes are difficult interpret. For example, in the study by Vilalta *et al*. [66] as discussed above, patients with ACS post TAVR had a high rate of mortality (37.3%) and even higher MACCE (46.7%) after 21 months of follow up. These rates were not stratified by treatment with PCI or medical therapy alone.

A recent study from France compared the characteristics and outcomes of patients that underwent PCI before versus after TAVR [67]. Over a ten-year period in more than 55,000 TAVR patients, 15% (n = 8613) had PCI within 90 days of the TAVR. The majority (8384 patients) had PCI before and only 229 patients had PCI within 90 days after TAVR. In a propensity-score matched analysis, all outcomes were identical except a non-significant trend for an increased cardiovascular mortality risk in the TAVR-first group likely explained by more urgent PCIs in this cohort.

## 8. Challenges in Coronary Access after TAVR

Coronary access after TAVR can be difficult as the stent struts of the valve sometimes interact and prohibit access to the left and right coronary arteries. This is a problem for all valve types, but more so for self-expanding valves where the stents protrude up as high as the tubular ascending aorta. Several small studies have evaluated the incidence and success rates in diagnostic coronary engagement post TAVR. For example, Chetchute *et al*. [68] evaluated 190 patients post CoreValve and found 97.9% patients underwent successful coronary engagement during coronary angiography. Among the cohort of 75 patients with independent adjudication of coronary engagement, the success rate was 96%. Overall, 91.2% of PCI attempts were successful, with a lower success amongst the adjudicated cases (81.6%). Zivelonghi *et al*. [61] assessed 66 consecutive patients after TAVR and found successful coronary engagement in 98%. Blumenstein *et al*. [62] demonstrated successful coronary engagement for diagnostic angiography in 97% of 34 cases. In each of these series, there was one failed attempt in a patient with a self-expanding valve. Htun *et al*. [69] showed 97% successful engagement of the left coronary artery and 90% for the right coronary artery in a review of 28 patients. In last 3 studies reviewed, all patients with successful coronary engagement had successful PCI. Boukantar *et al*. [64] retrospectively reviewed 550 patients after CoreValve implant and found 16 patients underwent post valve coronary angiography. They reported only 9/16 cases had completely successful angiography without a single case demonstrating selective engagement of both the left and right coronary arteries. Of note, the RCA was selectively engaged in only 2 of the 16 cases. PCI was successful in 6/7 cases within this group.

The recently published ALIGN-ACCESS study (Coronary Access After Transcatheter Aortic Valve Replacement with Commissural Alignment) evaluated the success of coronary angiography in 200 patients after receiving supra-annular Evolut R/Pro, supra-annular Acurate Neo, and intra-annular SAPIEN 3 [70]. The Evolut valves were implanted with intention for commissural alignment while just under half of the Acurate Neo implants attempted this alignment. Commissural alignment was at the discretion of the operator for the SAPIEN 3 valves. Coronary access was most successful in the SAPIEN 3 (95%) followed by aligned supra-annular valves (71%) and lastly misaligned supra-annular valves (46%) with *p *≤ 0.001. Commissural alignment was achieved in 85.9% of Evolut valves and 88.5% of intentionally attempted Acurate Neo valves. Cannulation of at least one coronary artery was not possible in 11% of misaligned supra-annular valves, 3% aligned supra-annular valves, and 0% in the SAPIEN 3 valves. Independent predictors identified in this trial of poor coronary engagement were implantation of a misaligned supra-annular valve, shorter sinus of Valsalva height, and THV to sinus of Valsalva relation.

There have been advancements in understanding ways to increase vessel engagement success. Techniques such as lower target implant depths for valve deployment and commissural alignment methods have been evaluated with potential of facilitating coronary engagement post-TAVR implant [71, 72]. New and future iterations of TAVR valves are also being designed with coronary engagement success in mind. These design iterations include targeted commissural alignment and larger stent strut cells in order to ease future coronary engagement [73].

Procedural techniques at the time of angiography may also increase diagnostic and procedural success rates. In cases of difficult RCA engagement post TAVR, shorter tipped catheters such as the FR4 or JR4 may provide greater chance of access success [74]. Further, the use of a guide catheter rather than diagnostic catheters allows for the utilization of guide wires and guide extension catheters which can help facilitate coronary engagement and allow for equipment delivery [75]. Similarly, certain techniques can be used to help engage the left main coronary artery after TAVR. Using a shorter tipped JL or FL (downsized by 0.5 or 1 size) catheter may increase coronary engagement success [74]. There have been case reports of operator success with the Ikari line of catheters for either coronary vessel [75]. Recently launched smart phone-based app dedicated to assist in coronary engagement post-TAVR can also be utilized for procedural planning or in real time to help enable the operator in using techniques to improve access success [76].

## 9. Conclusions

Obstructive CAD in patients undergoing TAVR is common. Studies on the optimal method for evaluating the significance of coronary stenoses as well as the timing of PCI in TAVR are ongoing. Coronary angiography post TAVR presents unique challenges, but advancements in valve technology and deployment techniques should improve coronary access over time.

## Abbreviations

ACS, Acute coronary syndrome; AS, Aortic stenosis; CAD, Coronary artery disease; CTA, Computed tomographic angiography; FFR, Fractional flow reserve; HR, Hazard ratio; iFR, Instantaneous wave-free ratio; MACCE, Major adverse cardiac and cerebrovascular events; MI, Myocardial infarction; OR, Odds ratio; PCI, Percutaneous coronary intervention; RCT, Randomized controlled trial; SAVR, Surgical aortic valve replacement; SS, SYNTAX score; TAVR, Transcatheter aortic valve replacement.
